# Correction to: Simulations to predict clinical trial outcome of bevacizumab plus chemotherapy vs. chemotherapy alone in patients with first‐line gastric cancer and elevated plasma VEGF‐A

**DOI:** 10.1002/psp4.13210

**Published:** 2024-07-23

Han, K., Claret, L., Piao, Y., Hegde, P., Joshi, A., Powell, J., Jin, J. and Bruno, R. (2016), Simulations to predict clinical trial outcome of bevacizumab plus chemotherapy vs. chemotherapy alone in patients with first‐line gastric cancer and elevated plasma VEGF‐A. *CPT Pharmacometrics Syst. Pharmacol*., 5: 352–358. https://doi.org/10.1002/psp4.12064

The first author of this paper, K. Han, should be listed as Kelong Han.

